# Ontogenetic Patterning of Human Subchondral Bone Microarchitecture in the Proximal Tibia

**DOI:** 10.3390/biology11071002

**Published:** 2022-07-01

**Authors:** Jesse R. Goliath, James H. Gosman, Sam D. Stout, Timothy M. Ryan

**Affiliations:** 1 Department of Anthropology and Middle Eastern Cultures, Mississippi State University, Mississippi State, MS 39762, USA; 2 Department of Anthropology, The Ohio State University, Columbus, OH 43210, USA; jgosman@buckeye-express.com (J.H.G.); stout.126@osu.edu (S.D.S.); 3 Department of Anthropology, Pennsylvania State University, State College, PA 16801, USA; tmr21@psu.edu; 4 Center for Quantitative Imaging, EMS Energy Institute, Pennsylvania State University, State College, PA 16801, USA

**Keywords:** functional morphology, bone microstructure, skeletal biology, growth and development, 3D imaging

## Abstract

**Simple Summary:**

The objective of this study is to explain the subchondral trabecular and the cortical ontogenetic changes that occur in the proximal tibia in both the medial and lateral condylar regions due to differential loadings associated with changing knee joint kinetics and body mass. The differential response of subchondral bone to changing mechanical loads during growth and development serves as a powerful tool to evaluate the significance of mechanical loading on subchondral bone morphology and joint development, and can offer insight into adult morphological variation for joint health.

**Abstract:**

High-resolution computed tomography images were acquired for 31 proximal human tibiae, age 8 to 37.5 years, from Norris Farms #36 cemetery site (A.D. 1300). Morphometric analysis of subchondral cortical and trabecular bone architecture was performed between and within the tibial condyles. Kruskal–Wallis and Wilcoxon signed-rank tests were used to examine the association between region, age, body mass, and each morphometric parameter. The findings indicate that age-related changes in mechanical loading have varied effects on subchondral bone morphology. With age, trabecular microstructure increased in bone volume fraction (*p* = 0.033) and degree of anisotropy (*p* = 0.012), and decreased in connectivity density (*p* = 0.001). In the subchondral cortical plate, there was an increase in thickness (*p* < 0.001). When comparing condylar regions, only degree of anisotropy differed (*p* = 0.004) between the medial and lateral condyles. Trabeculae in the medial condyle were more anisotropic than in the lateral region. This research represents an innovative approach to quantifying both cortical and trabecular subchondral bone microarchitecture in archaeological remains.

## 1. Introduction

The use of three-dimensional (3D) bone architecture for reconstructing the paleobiology of humans and other primates has become widespread in the field [1,2,3,4,5,6,7,8,9,10,11,12,13,14,15,16,17]. The effectiveness of bone, especially subchondral bone, for reconstructing behavioral and locomotive patterns depends on a better understanding of the relationships among bone structure, biomechanical loading, and behavior, as well as an understanding of the mechanical role of bone in various joints [18,19]. Adult bone morphology incorporates structural features established during ontogeny and is modified by biological factors and functional adaptive changes during maturation [20,21]. The response of bone to mechanical loading, especially during development, plays an important role in skeletal adaptation and determines much of adult bone morphology [22,23]. Experimental studies have demonstrated that the 3D arrangement of trabecular bone reflects variation in mechanical properties at specific anatomical locations [9,11,24,25,26,27,28,29]. Understanding the spatial specifics of ontogenetic processes during bone development, therefore, can offer insight into normal and pathognomonic morphological variation. This understanding also has implications for activity patterns, locomotion, and mechanical load within and between populations. Ontogenetic change in human and non-human bone has been a topic of considerable research [1,3,9,17,24,27,30,31,32,33,34,35,36,37]. In regard to bone microarchitecture, ontogenetic patterns provide major insight into the form and structure of bone. Growth is the most opportune time to modify the mass of the skeleton [38]. More specifically, growing bone exhibits the greatest functional responsiveness to mechanical stimulation, with tissue sensitivity diminishing rapidly once skeletal maturity is attained [39,40]. Bioarchaeologists who employ the principles of bone functional adaptation to the study of physical activity often analyze cortical and trabecular bone separately. However, little is known about the intimate intersection present in the subchondral bone, which consists of both cortical and trabecular architecture. Briefly, we will discuss the morphology and anatomy of subchondral bone.

### 1.1. Morphology and Anatomy of Subchondral Bone

A number of studies [19,41,42,43,44,45,46,47] have established that the anatomy of subchondral bone is highly variable. Duncan et al. [48] defines the subchondral plate as a zone which separates the articular cartilage from the marrow cavity and consists of two layers: the calcified region of the articular cartilage and a layer of lamellar bone. Müller-Gerbl [47] further defines “the subchondral zone” or “subchondral bone plate” as the bony lamella lying beneath the calcified zone of the articular cartilage. Depending upon the joint, this varies in thickness [49]. Several human studies [49,50,51] have found greater subchondral bone and plate density in the medial rather than in the lateral part of the tibial plateau. At places within the joint where the stress is greatest, the density is higher, the thickness is greater, and the vascularization is more strongly developed [18,47,52]. The trabeculae arising from this bony lamella are referred to as “supporting trabeculae” [49].

Here, “subchondral bone” is defined as both the subchondral cortical plate directly beneath the calcified cartilage of the articular cartilage and the underlying supporting trabeculae, referred to as subchondral trabecular bone (subarticular spongiosa). The biological interaction and mechanical mutual support make subchondral bone and cartilage a functional unit that cannot be separated [53,54]. Subchondral bone is a part of the osteochondral junction, which comprises the deeper non-calcified cartilage, calcified cartilage, and the underlying subchondral bone. The subchondral bone provides support and protection for its adjacent cartilage.

### 1.2. Subchondral Bone Loading and the Impact of Knee Joint Alignment

The mechanical integrity of the cartilage and its resistance to injury depends on its communication with the underlying subchondral bone [55,56]. Both structures, the cartilage and its supporting subchondral bone, have corresponding mechanical functions. The cartilage serves as the weight bearer and the subchondral bone serves as a structural support and shock absorber [45,48,57,58,59]. The subchondral bone absorbs a majority of the mechanical load transmitted by synovial joints [60]. Due to the greater stiffness and strength of the subchondral bone in comparison with the articular cartilage [61,62], it is generally established that the subchondral bone plays an important role in intra-articular load transmission [63,64,65,66] and that this bone strength increases with age [67]. The subchondral region exhibits the strongest architectural response to differences in joint loading regimes [68] and serves to maintain joint shape.

One crucial aspect of understanding this load in the proximal tibia is examining knee joint angle or alignment. The development of the knee angle shifts from bowlegged (varus) in infancy to knocked knees (valgus) in early childhood and stabilizes to a less valgus alignment as part of normal and physiological development [69,70,71,72,73,74]. However, the age ranges at which these phases come in children and adolescents have been found to differ across ethnic groups [69,71,72,75,76,77,78]. For example, [72] notes that stabilizing knee alignment can occur after the age of 10 years in Indian children. These age-related changes in limb alignment at the knee shift weight from the medial condyle to the lateral condyle and then back to the medial [79]. Because the subchondral bone absorbs a majority of the joint mechanical load, it has been argued that loading is the primary factor in explaining its orientation [80,81,82,83].

The biomechanical loading produced during early walking does differ from that of a mature gait, and differences between loads result in differential modeling in the lower limbs in young children and adults [84]. Given that microarchitectural changes in subchondral bone influence joint maintenance in later life [44,85,86,87,88], little research has been directed toward the structure of and variation in human subchondral bone during ontogeny with increasing body mass. Gosman et al. [40] suggests that a higher BV/TV in the lower limbs is influenced by load-bearing mechanical forces, which may be stronger on the medial condyle due to the anatomical positions of the distal femur and proximal tibia. The present study builds on prior research to better understand the complex nature of subchondral ontogenetic development with increasing body mass [3,34].

### 1.3. Aims and Hypotheses

This research examines subchondral bone microarchitecture changes during growth and development in subadult and adult skeletal remains associated with the Oneota (Norris Farms #36) archaeological population. More specifically, we aim to explain the trabecular and cortical tissue level age-related changes that occur in proximal tibia subchondral bone in both the medial and lateral condylar regions and assess whether age-related trends in the properties differ between the condyles. Age-related alterations in the structure and material properties of subchondral trabecular bone in the proximal tibia have only been investigated in a small number of studies [3,89,90,91,92]. Ding et al. [91] investigates normal age-related (16–85 years) changes in trabecular microstructural properties and demonstrates that the decrease in mechanical properties of trabecular bone in the proximal tibia with aging is a consequence of the loss of trabecular material. The study showed that bone volume fraction (BV/TV) decreased significantly with age; connectivity density (Conn.D) did not have a relationship with age; and degree of anisotropy (DA) increased with age. These age-related changes had the same trend and pattern for both the medial and lateral condyles of the tibia [90,91,93]. Gosman and Ketcham [3] found that in young adult individuals from SunWatch Village (16–20 years old), subchondral bone had a decrease in trabecular number (Tb.N) and DA, and an increase in BV/TV with age. Chen et al. [89] examines proximal tibia structural parameters in elderly Japanese populations, demonstrating a decrease in BV/TV and trabecular thickness (Tb.Th) with age in both women and men. It is important to note that comparisons between studies are difficult due to differences in populations, anatomical location, and experimental conditions.

We postulate that with the increase in body mass and refinement in adult gait, all subchondral bone morphometric parameters will be affected by age. Subchondral trabecular bone is expected to follow the same ontogenetic processes as other trabecular regions of the skeleton: a subsequent functional condensation of the underlying subchondral bone due to endochondral ossification with an increase in DA support [92]. In assessing subchondral bone at different ages during growth, it would be expected that subchondral bone becomes thicker with increasing age due to increasing Tb.Th. BV/TV will have a slight increase with decreases in Tb.N. These patterns have also been reported by other researchers examining trabecular ontogeny [3,36,83,92]. The ontogenetic changes seen in bone mass thickness and density occur with increases in load amount and duration and the changes seen in the distribution of trabeculae and their patterns are based on the direction of the load [81]. The remodeling of trabecular architecture includes an increase in BV/TV, an increase in Tb.Th, and a decrease in Tb.N [2,3,27,83]. Specifically, in subchondral trabecular bone, there will be an increase in BV/TV, DA and Tb.Th with age and a decrease in Conn.D, trabecular separation (Tb.Sp) and Tb.N with age. In the subchondral cortical plate, there would be an increase in thickness (Plate Ct.Th) with age. There is an expected increase in thickness due to similar mechanical forces affecting the underlying subchondral trabecular region [3,24].

Moreover, due to increasing mechanical load and the knee joint changing with development, we argue that there is a significant difference in subchondral trabecular bone and cortical plate morphometric parameters between the medial and lateral condyles with age. Multiple studies [47,50,90,91,94] show that the medial tibial condyle is stronger than the lateral condyle, and that in both regions the strength decreases rapidly with the distance from the surface. Because of this, we argue there will be greater thickness, Conn.D, BV/TV, and DA in the medial condyle than the lateral condyle. The lateral condyle will have greater Tb.Sp and Tb.N with less bone volume present.

## 2. Materials and Methods

### 2.1. Sample Composition

High-resolution computed tomography (HR-CT) scans of Norris Farms tibiae specimens from 31 individuals (12 males, 11 females, 8 subadults), ranging in age from 8 to 37.5 years old (average: 22.6 years), were used to examine subchondral trabecular bone and cortical plate ontogenetic changes. Only individuals with both subchondral trabecular bone and cortical plate were included in this study. The subchondral cortical plate first appears at 8 years old in this sample. The skeletal series was chosen for this study because of its cultural and biological homogeneity, high number of subadult individuals, extensive archaeological context, and excellent preservation [24,34]. The proximal tibia was chosen for this study because it is a skeletal region that is primarily controlled by axial compressive and tensile stresses and is commonly used in clinical and research studies of joint development and disease [34,95]. Norris Farms 36 site is a pre-contact cemetery from the central Illinois River Valley dating to approximately A.D. 1300; individuals are associated with the Oneota cultural tradition of village agriculturalists [96]. The burial population consists of 264 individuals, ranging in age from fetal to 50+ years, as determined by dental formation, sequences of epiphyseal closure, and age-associated changes [97]. All work was conducted via the analysis of HR-CT images.

### 2.2. CT Imaging

All skeletal analyses were performed using 3D digital models derived from HR-CT scans. All specimens were scanned at the Center for Quantitative X-Ray Imaging (CQI) at Pennsylvania State University using a Universal OMNI-X HD-600 Industrial High-Resolution X-ray CT system (Bio-Imaging Research, Inc., Lincolnshire, IL, USA). Gosman and Ketcham [3] found significant differences in trabecular bone properties between lower and higher resolution scans while analyzing micro-CT voxel dimension effects. For these reasons, proximal tibiae for each individual were scanned as two portions (medial and lateral condylar regions) for the best possible quality, and to reduce the effect of voxel size on bone properties. The resulting scans had voxel size ranging from 0.04 to 0.057 mm [36,98]. Regression analyses were run for each variable to test for significant influences of voxel size on trabecular properties. Statistically significant results were not found. Additionally, these differences in voxel size have little effect on the assessment of structures with relatively high thickness, such as cortical bone or trabeculae in humans [99].

Scanning involved foam mounting each specimen to stabilize the bone inside a thin-walled plastic tube with energy settings of 180 kilovolts (kV), 0.11 milliamps (mA), and 2800 projections; using a Feldkamp reconstruction algorithm, transverse cross-sectional slice images were collected for each tibia. Image reconstructions resulted in 1024 by 1024 pixel, 16-bit TIFF images [34,100]. Following scan data collection, the 16-bit images were converted to 8-bit binary TIFFs using ImageJ (v. 1.51f) [101] for the segmentation of regions of interest into trabecular and cortical volumes. Image stacks included between 860 and 3707 slices per bone (depending upon bone size and scan resolution). The voxel dimensions resulting from the scans were isotropic (i.e., voxels were perfect cubes). The voxel dimensions are reported in Table 1.

### 2.3. Volume of Interest (VOI) Placement and Size

**Figure 1:**
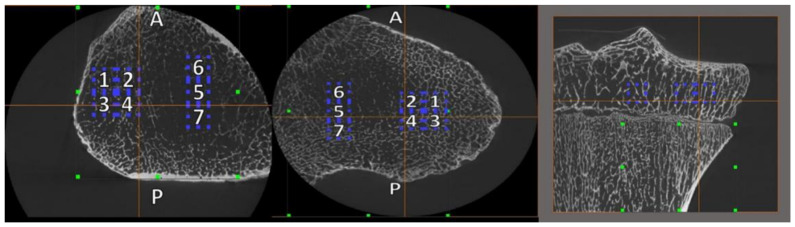
Proximal tibia VOI placements. Note: Left image—lateral condyle, transverse view; center image—medial condyle, transverse view; A—anterior; P—posterior; 1–4—VOIs within condyle; 5–7—intercondylar VOIs; right image—coronal view.

**Figure 2:**
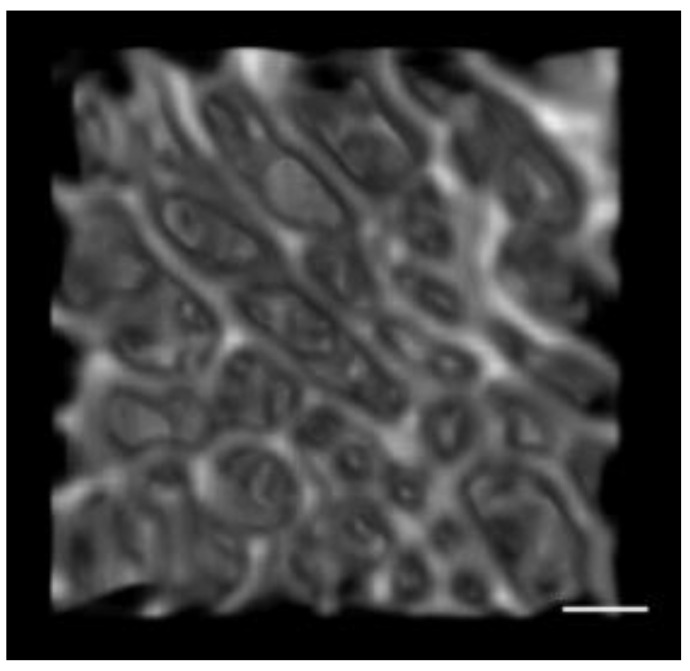
Example of an isolated trabecular (15.5 yr old) VOI using ImageJ. Note: Scale: 1 mm.

For analysis of the subchondral trabecular bone, four cubic VOIs were collected from the medial and lateral condyle of the proximal tibia using Avizo^®^ Fire 6.2 (Figure 1 and Figure 2). Data analyses were performed using Avizo^®^ Fire versions 6.2 and 8.1.1 (Thermo Fisher Scientific, Waltham, MA, USA), a data analysis and visualization software from FEI, and BoneJ, a plugin for bone image analysis in java-based ImageJ (v. 1.51f) [101]. BoneJ provides open-source tools for trabecular geometry and whole bone shape analysis [102,103].

VOI were positioned within and between tibial condyles within the epiphyseal region, just inferior to the proximal tibia’s contact area with the distal femur [11]. There are compressive forces in the proximal tibia during bipedal stance and locomotion [94]. By contrast, no direct compression is exerted upon VOIs between the condyles (i.e., central unloaded VOIs) during weight-bearing [24,47]. All intercondylar VOIs (VOIs 5, 6, 7) for both condylar regions were obtained as part of the imaging acquisition process but were not included in further analyses for this study. Multiple VOIs were used because the microarchitecture of trabecular and cortical bone is spatially mixed and is highly dependent on the volume of interest, position and size [3]. Previous researchers [14,104] have noted the importance of VOI size and location on trabecular properties, so the largest VOI possible was placed in order to ensure that each VOI was reflective of the structural variation between the joints. Because certain properties (connectivity and structure) are impacted by VOI size, each specimen’s VOI was adjusted to the individual [105] by using epiphyseal condylar breadth and the anteroposterior breadth of the proximal femoral metaphysis as the size standard. Each VOI size was calculated as 25% of the anteroposterior breadth of the proximal femoral metaphysis, resulting in cubic VOIs ranging in size from 4.0 to 8.178 mm, reflecting size increases in growth of the tibia across age [11]. VOI cube sizes are reported in Table 1.

### 2.4. Age, Sex, and Body Mass Estimation

All age-at-death and sex estimations for the Norris Farms 36 skeletal series were determined in a previous project [106]. Age-at-death for individuals in the samples was estimated according to standard methods for macroscopic skeletal age estimation [97,106]. Milner et al. [106] relied upon dental development [107,108,109,110] and epiphyseal closure [110] to estimate age-at-death in subadults, while adult ages were assessed via pubic symphysis morphology, endocranial suture closure, and auricular surface morphology [106]. Skeletal measurements used to calculate body mass estimates in this study were based on past research [100]. Body mass was estimated using [111] age-specific femoral head diameter equations. Body mass estimates are presented in Table 1.

### 2.5. Trabecular Bone Morphometric Parameters

For the analysis of the ontogenetic patterns in the subchondral trabecular bone, seven bone morphometric variables were quantified using BoneJ. The morphometric parameters used were indicators of bone mechanical properties, microarchitecture, and functional adaptation to loading history [112]. Descriptions for each parameter can be found in Table 2. BV/TV, DA, Tb.Th, and Tb.Sp were calculated directly from the high-resolution computed tomography (HR-CT) scans using Avizo^®^ Fire 6.2 and 8.1.1, and BoneJ. Derived structural variables, such as Tb.N and Conn.D, were calculated using BoneJ.

### 2.6. Cortical Masking

**Figure 3:**
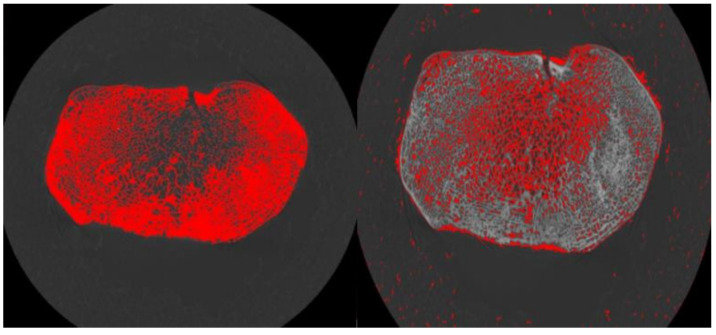
**Left** image: step one of separation script execution (highlighting bone). Note: bone material selected via threshold adjustments; 16.5-year-old tibia cross-section shown. **Right** image: step two of separation script execution (highlighting air space). Note: air space selected via a second set of threshold adjustments; 16.5-year-old tibia cross-section shown.

For the analysis of the subchondral plate, a cortical mask was necessary to ascertain subchondral cortical thickness properties (Plate Ct.Th). Proximal tibia scans for each individual were used to examine the thickness in each condylar region. Once a range of slices were visually identified as the region of interest for a particular bone plate, a truncated reconfirmed image stack comprising only those slices was imported into Avizo^®^ Fire version 8.1.1. for the masking of the cortical component of the bone image. This procedure was necessary in order to facilitate a later step in the data collection process to ascertain subchondral bone cortical plate properties. A custom script separated a region of interest into trabecular and cortical volumes. This script was similar to the dual-threshold technique developed by [119], which automatically segments cortical and trabecular compartments. In this study, grayscale threshold values were determined using a specimen-specific auto histogram-based thresholding method in ImageJ (*Optimise Threshold*) for the standardization of each specimen and to remove possible subjectivity. However, in some scans, bone and deeply embedded loess (soil) were not always sufficiently distinguished to allow for auto thresholding. These thresholds were adjusted and optimized by visual inspection. When necessary, the manual thresholding of a binary scan image was performed to ensure that no loess was included in the segmentation. This was accomplished by manually adjusting the threshold maximum and minimum values such that only bone was highlighted in the viewer window pertaining to each step in the script execution process (Figure 3). Once binarized (converted to a black and white image), a “set scale” using an individual scan voxel size (values in Table 1) was added for the quantification of the plate mean cortical thickness [100].

### 2.7. Statistical Analysis

Statistical analyses used SPSS version 27 (IBM SPSS Statistics 27.0 IBM, Armonk, NY, USA). Statistical analysis required that age-at-death estimates given as a range (e.g., 10 to 12 years) be converted to their mid-range value (e.g., 11 years). The VOIs associated with each region (lateral and medial) were averaged for each individual for analyses. All variables were tested for normality using the Shapiro–Wilk test. Of the ten variables, three (body mass, age, Tb.Sp) were not normally distributed and, therefore, nonparametric tests were used for all further analyses. The significance level was set at *p* ≤ 0.05 for all statistical tests. To test age-related influences on all morphometric parameters, the sample was divided into four age groups/categories based on sample demographics, tibial development, and previous growth studies [3,11,36,120]:

(1) Child (8–13.99 years, *n* = 6)

In humans, the trabecular structure of the tibia reaches an adult-like pattern (BV/TV, DA) typically at 8 years of age [3,11,36], with the modification of anisotropy in late childhood/prepuberty [3]. However, knee alignment may not be stabilized until after 10 years of age in some populations [72]. Thus, overall trabecular architecture appears to be optimized later in life [25,32,36,104,121]. At ages 8–13, the distal part of the tuberosity starts to ossify from one or more centers [122]. Individuals of this category have an increasing body mass, adult gait pattern, and presumably independent activity.

(2) Adolescent (14–19.99 years, *n* = 10)

Individuals in this category have increased body mass related to the pubertal growth spurt with a fully active adult lifestyle [92]. The proximal epiphysis begins to fuse at 13 years in females and 15.5 years in males, with later times extending to 17 years in females and 19.5 years in males [122].

(3) Young Adult (20–30.99 years, *n* = 4)

Individuals in this category have reached their peak attained bone mass and final attained height. Individuals have increased body mass with the cessation of growth [56].

(4) Middle Age (31–37.99 years, *n* = 11)

Individuals in this category continue to maintain their final attained height and body mass, but there are decreases in bone mass due to the endo-trabecular deficit of bone replacement during remodeling. Normal, age-related bone loss in trabecular bone begins to occur in men and women after age 30–35 [56].

Mean differences for each bone structural parameter (pooled sex and condylar regions) were tested across age categories by using an independent sample Kruskal–Wallis test and a Bonferroni correction post hoc test. Mean values for all variables across age category are in Table 3. Regional differences in parameters were tested via mean bone structural differences (sex-pooled) across condyle location using the related-sample pairwise Wilcoxon signed-rank test. The mean values for all morphometric parameters across the condyle locations are shown in Table 4. A Mann–Whitney U test was performed to examine the relationship between subchondral bone architecture and sex in the condylar regions. Finally, regression analyses were performed between body mass, all morphometric variables, and sex.

## 3. Results

### 3.1. Quantification of Subchondral Bone Structure by Age Category

Results for this section are condylar region- and sex-pooled.

- Child

The child age group was typified by having the lowest mean BV/TV (0.234), mean Tb.Th (0.282 mm), mean DA (0.6155), and mean Tb.N (0.826 mm^−1^) of the four age groups. This group had the highest mean Tb.Sp (0.858 mm) and mean Conn.D (4.132 mm^−3^) and thinnest mean Plate Ct.Th (0.887 mm).

- Adolescent

The adolescent age group was typified by an increase in Tb.Th (0.3192 mm), BV/TV (0.277), DA (0.6539), and Plate Ct.Th (1.1535 mm) when compared to the child age group. There was a general decline in Conn.D (3.1444 mm^−3^).

- Young Adult

The young adult age group was typified by having the highest mean BV/TV (0.290), mean Tb.Th (0.332 mm), mean Tb.N (0.8733 mm^−1^), and DA (0.686). There was a decline in Conn.D (2.4853 mm^−3^) and an increase in Plate Ct.Th (1.599 mm).

- Middle Age

**Figure 4:**
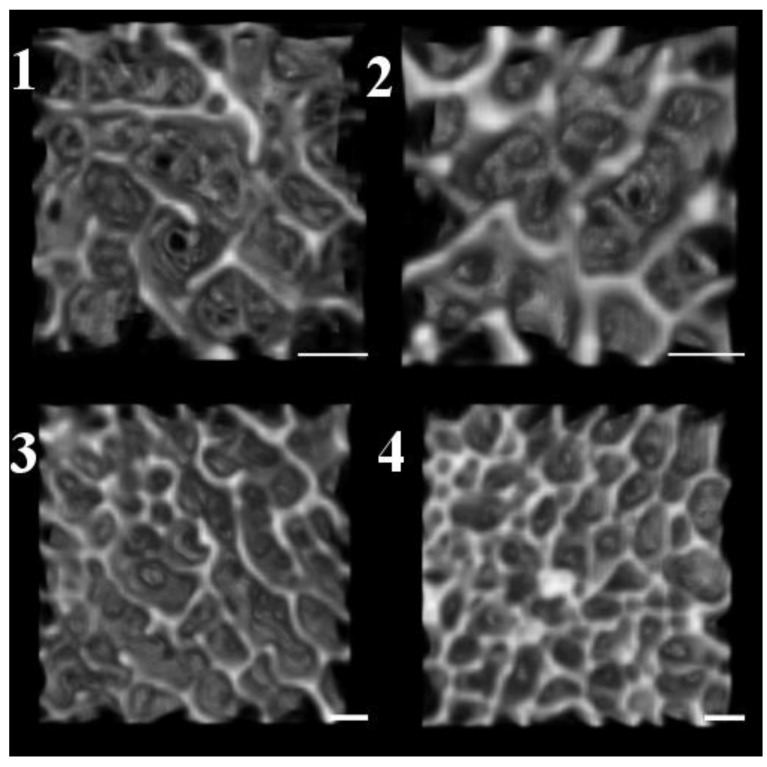
Volume of interest visual representation for each age category. **1** = Category 1 (9-year-old unknown sex); **2** = Category 2 (16-year-old male); **3** = Category 3 (26-year-old male); **4** = Category 4 (37.5-year-old male). Note: Scale: 1 mm. All images from VOI 2.

The middle age group was typified by a decline in BV/TV (0.2747) and Tb.Th (0.3158 mm) from the young adult group. The Plate Ct.Th (1.644 mm) was highest in this group. A VOI visual representation for each age category is provided in Figure 4.

**Figure 5:**
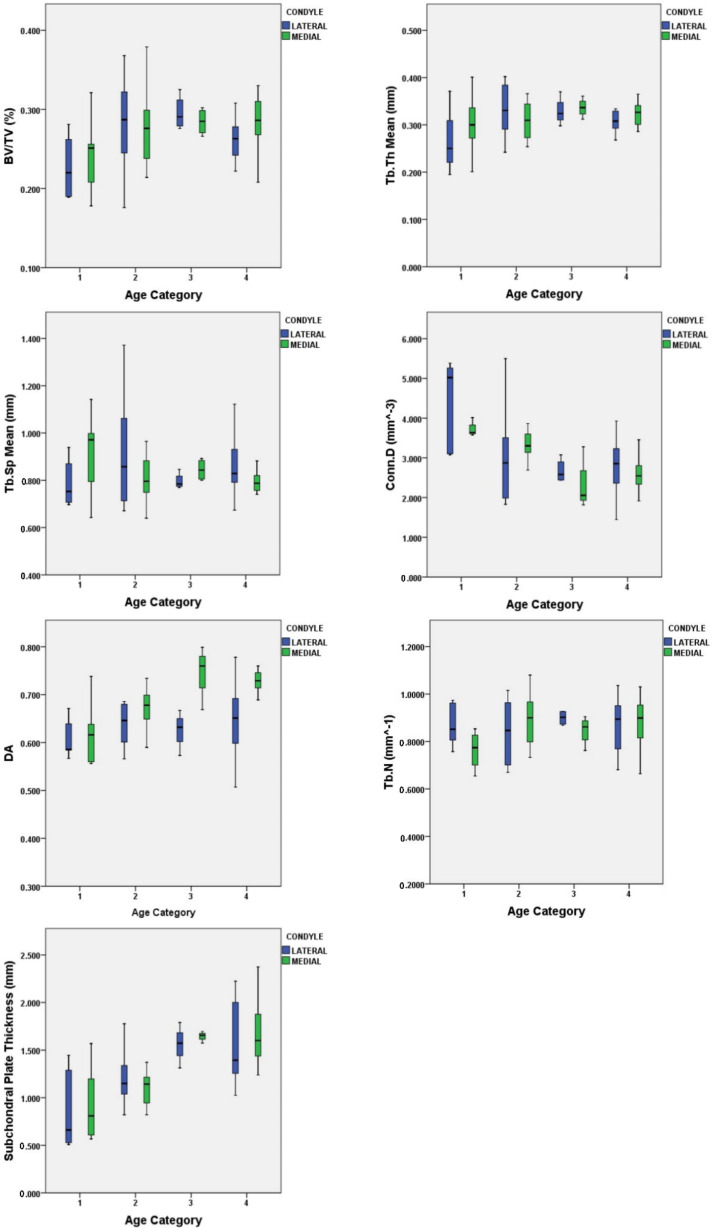
All morphometric parameters by condylar region across age categories.

When comparing age categories (condylar region- and sex-pooled), BV/TV was higher in Age Category 3 (20.0–30.99 years) compared to Age Category 1 (8.0–13.99 years). BV/TV increased with age from childhood to adult. DA was also greater in Age Category 4 (31.0–37.99 years) compared to Age Category 1. Trabecular subchondral bone became more anisotropic with the adult form. Conn.D was less in both Age Categories 3 and 4 compared to Age Category 1. A decline in overall trabecular connectivity density was present in both adult categories. Plate Ct.Th was greater in the Age Categories 3 and 4 (20.0–37.99 years) when compared to Age Categories 1 and 2 (8.0–19.99 years). This trend was present also between Age Categories 2 and 3. This represents an increase in Plate Ct.Th with age (Figure 5; Table 5 and Table 6).

### 3.2. Condyle Differences

The condylar regions only statistically differed in DA, with the medial condylar region being more anisotropic. On average, the medial condyle had a higher mean BV/TV (0.275 mm), Tb.Th (0.317 mm), DA (0.694), and thicker Plate Ct.Th (1.339 mm), while the lateral condyle had higher mean values of Tb.Sp (0.853 mm) and Conn.D (3.08 mm^−3^).

Pairwise related-sample Wilcoxon signed-ranks tests were performed on all morphometric variable means comparing the medial and lateral condylar regions. In the subchondral cortical plate, slight thickening was noted in the medial condyle, but there was no significant difference (*p* = 0.638) found. In regard to the subchondral trabecular bone, only DA significantly differed (*p* = 0.04) between the medial and lateral condylar regions (Table 7). The Wilcoxon signed-rank test revealed that the medial condyle ranked higher than the lateral condyle in the majority of paired cases for DA. Overall, the medial condyle had a larger mean value for BV/TV compared to the lateral. The lateral condyle had a larger mean value of Tb.Sp and Conn.D.

As predicted, with increasing body mass, age and condyle variation did occur in the subchondral trabecular bone and cortical plate. However, not all morphometric variables rejected the null hypothesis of no change with age and location. These results indicate that statistically significant differences between groups (*p* < 0.05) only occurred in BV/TV, Conn.D, DA, and Plate Ct.Th with age. Additionally, only DA significantly differed (*p* = 0.04) between the medial and lateral condylar regions. The regression results showed that with increasing body mass there was no change in morphometric variables except for a sharp decrease in Conn.D (*p* < 0.001).

**Figure 6:**
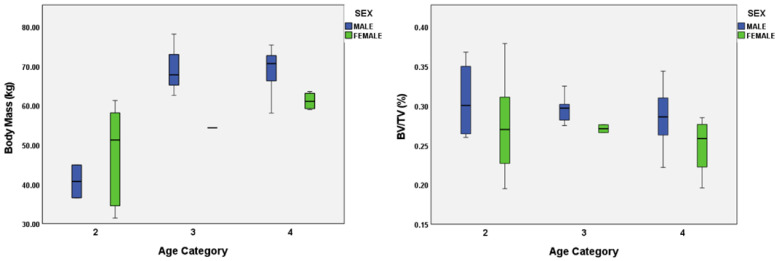
Body mass and bone volume fraction sex differences across age.

Sex was also examined as a possible variable in the later age categories. Statistical differences (*p* < 0.05) were found in body mass and BV/TV (Table 8). Overall, males had a higher mean BV/TV (i.e., more bone tissue) and greater body mass in the later age categories (Figure 6). However, it is important to note the sample sizes were small, especially as Category 3 only had one female.

## 4. Discussion

### 4.1. Characteristics of Subchondral Bone and Plate Ontogeny

Our results indicate that the human skeleton optimizes its microarchitecture via elaborate adaptations to mechanical loading during growth and development. With increasing body mass, the subchondral bone increases in BV/TV with age. A decline in overall Conn.D. was present in both adult categories. There was also an observed increase in DA. As a consequence of aging and the decline in Conn.D, the aging trabeculae seemed to align more strongly in the primary direction, becoming more anisotropic. Highly anisotropic trabecular bone is thought to signify a locomotor pattern that restricts joint mobility to a particular direction, whereas a more isotropic trabecular structure is considered to signal locomotor behavior involving greater joint mobility [123,124]. DA reflects consistent joint loading and, by extension, locomotor repertoire variability [124,125,126,127].

As noted, BV/TV increased with age from childhood to the adult stage and then remained constant in the middle age category. Comparing these cortical and trabecular bone quantifications with other proximal tibia subchondral ontogenetic studies, some general similarities and differences were found. Our results are similar to [3]. They noted in young adult individuals from the Fort Ancient site of SunWatch Village (16–20 years old) that subchondral bone had an increase in BV/TV with age and a decrease in Tb.N. However, our results differ from [91]’s research. The observations of [91] were geared toward much older individuals than the present study. The majority of this study’s samples fit in [91]’s young age range (16–39 yrs). In their younger individuals, [91] found no major changes in any morphometric parameters, but noted that BV/TV and Tb.Th decreased significantly after the age of 60. They also found that all the microarchitectural properties from the medial and lateral condyles had the same age-related trends. Possible factors for the differences seen between our study and [91]’s include secular change (biological generational changes), activity pattern differences, and genetic differences that could exist between a modern medical sample and our archaeological sample population. Moreover, [44,89] focused on a much older population with Japanese subjects ranging in age from 57 to 98 years old and noted that trabecular bone mineral density, BV/TV, and Tb.Th decreased between the middle-aged and elderly groups for both men and women. Our oldest age category (Category 4) individuals were still much younger than this Japanese sample, and retained stronger bone microarchitecture in relation to volume and thickness, emphasizing the influence of age in examining these structural properties.

In this study, there was an increase in Plate Ct.Th with age. Age-related plate thickness has been found in non-human studies [57,128], but the thickening of the subchondral bone plate has also been associated with the onset of osteoarthritis [44,129,130,131,132,133]. Because of its relatively greater stiffness and strength in comparison with the overlying cartilage [63,134,135], the subchondral plate is generally believed to play an important role in juxta articular load transmission. This appears to be the result of the greater potential for modelling trabecular tissue during later stages of development (and into adulthood perhaps), once the modelling of external bone shape has slowed/ceased [136].

### 4.2. Microarchitecture in Relation to Locomotion

Since both cortical bone and trabecular bone respond to changes in loading patterns, the response of bone structure to early irregular loading and then to more predictable loading during late childhood provides a unique morphological indicator of development in mature and stable gaits. With increasing age, [11] argues that mean DA converges at higher values and becomes less variable across the distal tibia. Our study reiterates this, as well as the fact that as maturation occurs the subchondral trabecular bone becomes more highly oriented in the longitudinal direction across the proximal tibia condylar regions [11]. It is also noteworthy that the most substantial increases in muscle mass occur after the pubertal growth spurt [137], mediated by an increase in growth hormones, and after linear growth has ceased. The implication of this is that the morphological shape changes observed during late adolescence/early adulthood are mainly accepted by internal structures [136].

### 4.3. Body Mass and Sexual Dimorphism

This study illustrates some sexual dimorphic differences in body mass and bone volume in the later age categories. However, due to the limitations of the dataset, no further analyses could be conducted. When comparing our results to other studies, there was not a clear agreement regarding sexual dimorphic changes in trabecular microarchitecture. Chen et al. [89] showed that men had higher BV/TV and lower Tb.Sp in elderly groups compared to women. Eckstein et al. [138] compared sex differences in trabecular bone microstructure across multiple skeletal sites and found males had thicker trabeculae, higher connectivity, and a higher DA in the femoral trochanter, but these results were not found in other skeletal sites in the same sample. Beresheim et al. [1] found no sex differences in any of the bone microstructure variables when examining thoracic ribs from a 15–17th century archaeological collection while [8] found multiple sex differences, but the patterns were not consistent across volumes of interest. Doershuk et al. [139] also found no clear pattern of dimorphism in the humerus or femur, and [16] found in the foot that with increasing body size there was no change in BV/TV or Tb.Th and a substantial decrease in Conn.D. Moreover, [8] argues that sex and body mass influences vary greatly. We postulate that this divergence in results is due to variation in the bones analyzed, sample populations, and differential loading affected by body mass.

### 4.4. Implications of this Study

The results of this research bolster previous findings by other studies of trabecular bone local responses to changes in loading patterns. The loss of tissue during infancy may be essential for developing a highly oriented structure that can resist loads efficiently with minimal bone mass. This also provides greater phenotypic plasticity and may be a response to developing postural and locomotor loads [30]. It is expected that both trabeculae and overall bone shape probably respond in tandem to mechanical loads during ontogeny [140], but that microstructural properties may continue after the adult shape has been attained. Additionally, these results suggest that subchondral bone microstructural properties are remarkably heterogenous.

### 4.5. Limitations

The most significant potential limitation to this study is the size and positioning of the volumes of interest. Previous analyses have clearly shown significant variation in bone structure within a single bone [13,36]. However, this study provides an alternative approach by positioning multiple volumes throughout the epiphyseal region with the idea of characterizing structure across the entire region. Moreover, the VOIs created were scaled to the size of each individual. The use of multiple volumes has been successful in previous analyses [141], but presents a challenge in comparing different bones with distinctly different shapes and sizes [13].

Understanding the developmental and morphological variation that exist in humans can help better define stress and lifestyle in past populations. However, there are inherent assumptions in interpreting growth and development in archaeological populations, including biological uniformitarianism, stationary populations, and the ability to determine accurate age estimates from skeletal material [142,143]. There are also biases in sample size, aging methodology, sex estimation, and preservation status that need to be addressed when performing analyses. By comparing modern growth standards with archaeological samples, we are comparing the growth of children who died to that of healthy living children from populations known to have had secular changes in recent decades. Additionally, it is difficult to determine if the growth of children in the archaeological record accurately reflects the growth of those who became adults.

Moreover, there are still concerns regarding making interpretations based solely on subchondral bone, such as what role articular cartilage plays in initial development, and whether we should examine these regions of the body as a functional joint–subchondral bone unit or as separate components.

## 5. Conclusions

Age-related changes in mechanical loading have varied effects on subchondral bone morphology within the proximal tibia. The nature of the structural response to mechanical stimuli may also provide valuable information about the relationship between joint disease and bone microstructure, especially in the weight-bearing skeleton. Behavioral reconstruction using subchondral bone structure in archaeological populations requires a fundamental understanding of the link between ontogenetic changes in bone architecture and the mechanical loads experienced during locomotion and other behaviors. This study provides a better understanding of the complexities of tissue level growth dynamics in the proximal tibia and provides a rare opportunity to study the effects of childhood bone growth on subchondral microstructural organization, which may have effects on the mechanical properties of bone well into adulthood. The trends highlighted in the current study provide important baseline information that can be used in future comparative studies of subchondral bone growth. This is important in both archaeological and orthopedic contexts to further clarify the mechanical sensitivity and functionally adaptive nature of subchondral bone. It is clear that new methods for detecting variance in bone morphology must be added to pre-existing ones to refine our understanding of the relationship between behavior, loading environment, function, and skeletal response.

## Figures and Tables

**Table 1:** Sample composition.

ID	Estimated Age (Years)	Age Category	Sex	Body Mass Estimation (kg)	Voxel Size (mm)	VOI Cube Size (mm)	VOI Length (# of Slices)
1	8	1	U	17.182	0.04	4.72	119
2	9	1		26.786	0.05	4.4	89
3	9	1		20.152	0.04	5.16	130
4	9.5	1		12.802		4	101
5	10.5	1		22.475	0.05	5.5	111
6	11	1		20.481		5.7	115
7	15	2		33.687		5.6	113
8	15.5	2	M?	36.562		6.55	132
9	16	2	F	34.549		6.2	124
10	16	2	M	44.901		4.35	88
11	16	2	U	39.148		6.15	124
12	16.5	2	F?	31.407		6.3	127
13	18	2	F	49.611	0.056	5.684	103
14	19.5	2		58.139	0.057	5.015	89
15	19.5	2	F?	61.282	0.056	6.322	114
16	19.5	2	F	52.893		6.438	116
17	21.5	3	M	62.587	0.057	7.772	137
18	22.5	3		78.169	0.056	8.178	147
19	26.5	3		67.804		8.12	146
20	27.5	3	F	54.349		6.148	111
21	32.5	4		58.970		6.902	124
22	32.5	4		59.480	0.057	6.844	121
23	32.5	4	M	65.678		5.684	101
24	32.5	4		58.092	0.056	6.554	118
25	32.5	4	F	62.670		6.264	113
26	32.5	4	M	73.941		8.294	149
27	32.5	4		70.679		6.438	116
28	35	4	F	63.570		6.728	121
29	37.5	4	M	71.477	0.057	7.328	130
30	37.5	4		66.862	0.056	7.192	129
31	37.5	4		75.391		7.598	137

All measurements are in millimeters (mm). M = male; M? = possible male; F = female; F? = possible female; U = unknown; VOI = volume of interest. # = number.

**Table 2:** Description of bone morphometric variables.

Bone Morphometric Variable (Unit)	Description	References
Bone Volume Fraction (%)	Ratio showing what proportion of a volume is comprised of trabecular bone/bone tissue	[105,113]
Trabecular Thickness (mm)	Measure of the average thickness of trabecular struts	[105,114]
Subchondral Cortical Plate Thickness (mm)	Mean cortex thickness	
Trabecular Separation (mm)	Mean distance between trabeculae	[105,114,115]
Trabecular Number (mm^−1^)	Ratio of bone volume fraction to trabecular thickness, a measure of the number of traversals across a trabecular or solid structure	[105]
Connectivity Density (mm^−3^)	Measure of the ‘connectedness’ of trabeculae to one another within the VOI	[116,117]
Degree of Anisotropy (unitless)	Measure of the directional orientation of trabeculae, ranging from 0 (fully isotropic) to 1 (fully anisotropic)	[105,118]

**Table 3:** Mean statistics for all variables by age category. Note: body mass is sex-pooled, while the other parameters are region-pooled (i.e., both condyles).

Variables (Unit)	Age Category	*n*	Mean (Standard Deviation)
Bone Volume Fraction (%)	1	11	0.234 (0.04)
	2	20	0.278 (0.06)
	3	8	0.290 (0.02)
	4	22	0.274 (0.03)
Trabecular Thickness (mm)	1	11	0.282 (0.07)
	2	20	0.320 (0.05)
	3	8	0.333 (0.02)
	4	22	0.316 (0.02)
Trabecular Separation (mm)	1	11	0.843 (0.15)
	2	20	0.859 (0.19)
	3	8	0.820 (0.05)
	4	22	0.832 (0.11)
Connectivity Density (mm^−3^)	1	8	4.133 (0.95)
	2	20	3.144 (0.84)
	3	8	2.485 (0.51)
	4	21	2.680 (0.57)
Degree of Anisotropy (-)	1	11	0.616 (0.06)
	2	20	0.654 (0.05)
	3	8	0.687 (0.08)
	4	22	0.684 (0.07)
Trabecular Number (mm^−1^)	1	11	0.826 (0.10)
	2	20	0.870 (0.12)
	3	8	0.873 (0.05)
	4	22	0.872 (0.11)
Subchondral Cortical Plate Thickness (mm)	1	11	0.888 (0.39)
	2	20	1.154 (0.24)
	3	8	1.600 (0.16)
	4	22	1.645 (0.39)
Body Mass (kg)	1	6	19.979 (1.93)
	2	10	44.219 (3.40)
	3	4	65.727 (4.99)
	4	11	66.074 (1.85)

**Table 4:** Mean statistics for all morphometric variables by condyle region. Note: lateral condyle = 1; medial condyle = 2.

Variable (Unit)	Condyle	Mean	Standard Deviation
Bone Volume Fraction (%)	1	0.2651	0.0468
	2	0.2753	0.0449
Trabecular Thickness (mm)	1	0.3091	0.0505
	2	0.317	0.0404
Trabecular Separation (mm)	1	0.8538	0.1623
	2	0.826	0.1108
Connectivity Density (mm^−3^)	1	3.0826	1.0485
	2	2.9423	0.6384
Degree of Anisotropy (-)	1	0.634	0.0577
	2	0.6936	0.0623
Trabecular Number (mm^−1^)	1	0.8635	0.1083
	2	0.8634	0.1066
Subchondral Cortical Plate Thickness (mm)	1	1.300	0.4668
	2	1.3387	0.4425

**Table 5:** Age category independent sample Kruskal–Wallis test.

Statistical Test	BV/TV (%)	Tb.Th (mm)	Tb.Sp (mm)	Conn.D (mm^−3^)	DA (-)	Tb.N (mm^−1^)	Plate Ct.Th (mm)	Body Mass (kg)
Kruskal–Wallis	8.752	4.711	0.547	17.345	10.934	0.515	26.861	23.669
df	3	3	3	3	3	3	3	3
*p*-value	0.033	0.194	0.908	<0.001	0.012	0.916	<0.001	<0.001

**Table 6:** Kruskal–Wallis post hoc test for significant age category comparisons.

Variable (Unit)	Age Category Comparison	*p*-Value
BV/TV (%)	1 vs. 3	0.040
Conn.D (mm^−3)^	1 vs. 3	0.003
	1 vs. 4	0.003
DA (-)	1 vs. 4	0.011
Plate Ct.Th (mm)	1 vs. 3	0.004
	1 vs. 4	<0.001
	2 vs. 3	0.040
	2 vs. 4	0.030

**Table 7:** Pairwise related-sample Wilcoxon signed-rank test comparing condylar parameter differences.

Variable (Unit)	Mean Rank (Negative)	Mean Rank (Positive)	Z	*p*-Value
BV/TV (%)	18.75	13.03	−0.649	0.516
Tb.Th (mm)	15.13	14.03	−0.490	0.624
Tb.Sp (mm)	13.35	13.65	−0.051	0.959
Conn.D (mm^−3^)	15.31	11.69	−0.597	0.551
Tb.N (mm^−1^)	13.24	16.45	−0.501	0.616
DA (-)	10.93	15.69	−2.881	0.004
Plate Ct.Th (mm)	15.70	12.13	−0.470	0.638

**Table 8:** Mann–Whitney test comparing parameters between sexes.

Statistical Test	BV/TV (%)	Tb.Th Mean (mm)	Tb.Sp Mean (mm)	Conn.D (mm^−3^)	DA	Tb.N (mm^−1^)	Plate Ct.Th (mm)	Body Mass (kg)
Mann–Whitney U	159.0	217.0	268.5	304.0	261.5	178.0	188.0	26.0
Z	−2.309	−1.034	0.099	1.183	−0.055	−1.891	−1.671	−2.462
*p*-value	0.021	0.301	0.921	0.237	0.956	0.059	0.095	0.013

## Data Availability

The data that support the findings of this study are available from the corresponding author upon reasonable request.
